# People's Banks

**Published:** 1898-09-17

**Authors:** 


### Modern Sociology.

### PEOPLE'S BANKS.

### I.—Schultz's Credit Associations.

It is often said that the pawnbroker is the banker of the poor. The statement has a certain amount of truth in it; and it is worth while to inquire why the pawnbroker does not enjoy in popular esteem the same position as the banker, and why it is looked upon as reprehensible, or at least pitiful to have dealings with him. One reason is the usurious rate of interest he charges, 25 per cent. or more, as against the ordinary bankers' 3 to 5. A man who has borrowed any considerable sum at this rate has but little chance of ever getting out of debt. In the second place, the pawnbroker takes into his own keeping articles belonging to the borrower as a pledge of repayment or compensation in case of default. During the existence of the loan the borrower is deprived of the use of the articles pledged, and when, as is often the case, the articles are necessaries or comforts of life—above all, when they are instruments of production—the borrower is poorer on the one hand by as much as he is richer on the other. Credit given for the obtaining articles of consumption is an almost unmixed evil; while credit given for articles of production is often a great economical benefit. Bankers' credit is generally given for purposes of production; the pawnbroker's is given for articles of consumption, and the *vox populi*, in this case at least speaking wisely, instinctively condemns the one and approves the other.

But why should a poor man not have a banker as well as a richer one? It is in this country very difficult for a man to get the first start in business, though it may be easy enough for him to get foothold for the second and succeeding ones. In Scotland the cash-credit system has done much for ambitious young men of good character, who could find one or two friends with sufficient belief in their industry and capacity to guarantee a bank account for them; but abroad, especially in Germany and Italy, the system of people's banks has been carried much farther than it has here, with results that have proved how beneficial good banking facilities, managed on sound business principles, may be to the poorer classes.

The pioneer country was certainly Germany; whether the pioneer himself was Schultz or Raffeisen is undecided; for both started their work about the same time, and both accomplished great things. Schultz (known from the name of his birthplace as Schultz-Delitsch) has carried on operations on a larger scale, so we will speak of him first. He was a provincial judge, and as such had come into contact with many of the unfortunate among the poor when, in 1850, he started his first "Credit Association." These associations are primarily savings banks, though they fulfil a larger function than the institutions known by that name here. In forming a bank, the first thing is to secure a capital of guarantee. Schultz started with a small amount of borrowed money; but for the most part he depended upon the contributions of the shareholders, who were encouraged to join the association by having a prior claim to have money lent them by it. In the original association, the share was fixed at £30, and no one was allowed to have more than one, as it was not intended to put the institution into the hands of capitalists. A member was not expected to pay up his share in full before he began to participate in the banking facilities of the association; he could at once borrow on any reasonable security. Schultz's chief desire was to encourage the shareholder to save; that was why the share was so high priced; but it was no less an advantage that he could, on the strength of his share, obtain an advance on what he possessed. Loans were not, however, cheap; 12 to 14 per cent. was charged at first, and even now the ordinary rate is 6 to 8 per cent. But this seemed cheap to people who had been in the habit of paying to the usurers 100 to 200 per cent. To prevent the loans being a mere form of indebtedness, never meant to be liquidated, the term for which money was given was three months; and though sometimes a renewal for three months longer was granted this was not encouraged. That the Schultz banks met a felt want may be guessed from the growth of them. Begun in 1850 with a single institution, possessed of only a small capital (£555), there are now about a thousand co-operative banks of this type, doing business to the amount of about £130,000,000. Perhaps these associations have not fully answered to the intention of their founder. They have, indeed, done well for the shareholders; and as these, holding only one share each, may very often be far from rich men, this is gratifying. People with only a small capital require a larger return for their money in order to make them feel that it is worth their while to save at all. Still, when we hear of some of the Schultz banks paying 12, 14, even 20 and 30 per cent., we begin to think that those who borrow from these banks cannot be so well served, all the more that some of the associations have departed from the wise rule of the founder, that loans should be granted to members only. This extension of the borrowing community has not always, however, led to profit-making. It has, added to the fact that the officials of the associations are paid partly by commissions on the business transacted through their branches—gross business done, not profits earned—caused loans to be given on insufficient security, or such as could not easily be realised. This has caused one or two of the associations to become bankrupt. Another fault is that the loans, being granted for such short periods, are of no use to a class to whom they would be a decided benefit—the small farmers and peasant proprietors who are so numerous on the Continent. If a farmer gets a loan for the purchase of seed, he cannot be in a position to repay it for at least six months; if he borrows in order to buy some expensive piece of machinery, it is possible that he may require longer credit still. The Schultz institutions cannot, therefore, be regarded as having fully occupied the field open to people's banks. But they have proved very useful to the small shopkeeping class, who often have to pay for goods which they have not yet sold, or, having sold, have not in turn been paid for; to the better class of artisans, and the like. Above all, they have encouraged people to save by giving them a profit on their savings which makes the 2½ per cent. of our Government savings bank look small indeed.

